# Supplementary material for: The causal relationship between the human gut microbiota and pyogenic arthritis: a Mendelian randomization study
Source: Front Cell Infect Microbiol. 2024 Nov 26;14:1452480. doi: 10.3389/fcimb.2024.1452480 (PMC11629706; doi:10.3389/fcimb.2024.1452480)
Supplement: Supplementary file 1 [file DataSheet1.zip › mendelian test/ebi-a-GCST90027664.csv_leave_one_out.pdf]

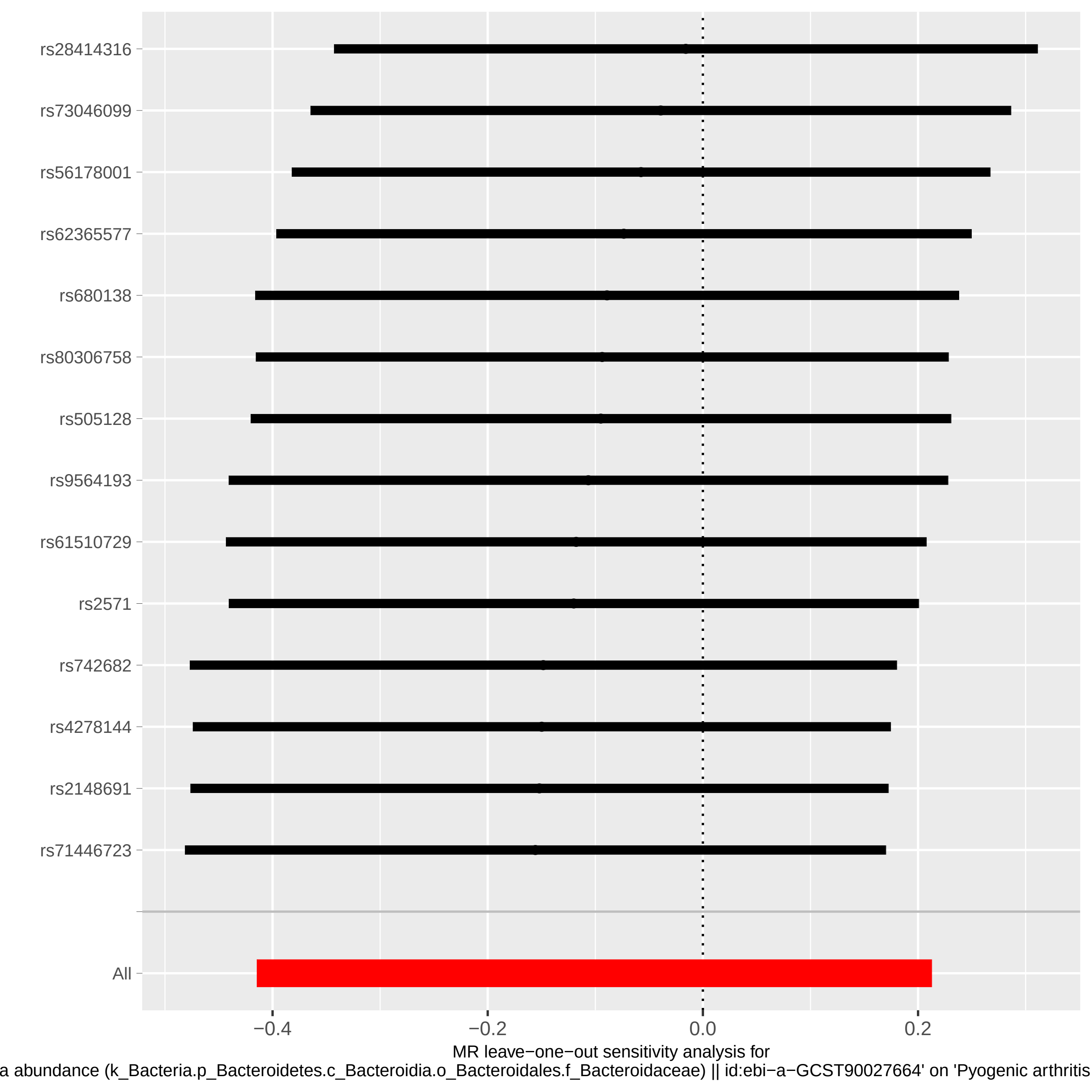

rs28414316
rs73046099
rs56178001
rs62365577
rs680138
rs80306758
rs505128
rs9564193
rs61510729
rs2571
rs742682
rs4278144
rs2148691
rs71446723
All
−0.4
−0.2
0.0
0.2
MR leave−one−out sensitivity analysis for
a abundance (k_Bacteria.p_Bacteroidetes.c_Bacteroidia.o_Bacteroidales.f_Bacteroidaceae) || id:ebi−a−GCST90027664' on 'Pyogenic arthritis
